# A wide spectrum of fastidious and ampicillin-susceptible bacteria dominate in animal-caused wounds

**DOI:** 10.1007/s10096-016-2667-z

**Published:** 2016-05-19

**Authors:** O. Gustavsson, A. V. Johansson, H.-J. Monstein, L. E. Nilsson, A. Bredberg

**Affiliations:** Department of Clinical Microbiology, Linköping University, Linköping, Sweden; Innlandet Hospital Trust, Lillehammer, Norway; Clinical Microbiology, University Hospital, 58185 Linköping, Sweden; Department of Clinical and Experimental Medicine, Faculty of Medicine and Health Sciences, Linköping University, Linköping, Sweden; Lund University, Lund, Sweden

## Abstract

The main purpose of this study was to assess the actual occurrence of Gram-negative oxidase-positive bacteria (GNOP) in human wounds caused by animals, mostly cat and dog bites and scratches, and with signs of infection. We report a prospective series of 92 wound samples. Routine culturing was combined with a procedure optimised for fastidious GNOP. All GNOP isolates were identified by 16S rDNA sequencing to the species level. We observed a more prominent role of GNOP, including at least 30 species mostly in the families Flavobacteriaceae, Neisseriaceae and Pasteurellaceae, and less of *Staphylococcus aureus* and streptococci. The antibiotic susceptibility pattern was investigated, as GNOP are associated with sudden onset of serious infections, making an early decision on antibiotic treatment vital. All GNOP isolates judged to be clinically relevant displayed susceptibility to ampicillin and meropenem, but resistance to oxacillin, clindamycin and gentamicin was frequent. Our findings emphasise the need to cover GNOP as recommended in guidelines, and not only common wound pathogens, when treating an animal-caused wound.

## Introduction

It is known that a number of bacterial species can often be recovered from an animal-caused wound, with common human wound pathogens such as *Staphylococcus aureus*, streptococci and anaerobes, as well as Gram-negative oxidase-positive bacteria (GNOP) seldom observed in other wound settings and apparently representing the animal’s oral flora [1–4]. Most of the published data are from the 1970s to 1990s, with a focus on Pasteurellaceae among the GNOP. In the two last decades, cultivation and species identification methods have become refined. Therefore, we reasoned that the actual clinical role of GNOP may be under-estimated, and that the extent of resistance to the antibiotics commonly used is not fully known. An improved knowledge on the spectrum of GNOP appearing in animal-caused human wounds can help us decide how to use pre-emptive (prophylactic) treatment and how to formulate empiric antibiotic treatment guidelines for wounds with signs of infection. These considerations seem to be clinically relevant, considering that GNOP infections can have an early, sudden and serious onset, sometimes even more marked than what is usually seen with *S. aureus* and other common wound pathogens [5–7].

The main aim of this study was to assess the actual incidence during half of the year 2009 of GNOP in animal-caused wound infections, by using a protocol with special media and a prolonged incubation time. We sought identification of all GNOP isolates at the species level with 16S rDNA sequencing. A parallel standard clinical routine culturing was used for assessment of the frequency of common wound pathogens. A second aim was to determine the extent of resistance to commonly used antibiotics, by performing an in vitro minimum inhibitory concentration (MIC) analysis of all GNOP isolates.

## Materials and methods

### Wound samples

All samples with information indicating an infected wound caused by an animal submitted during April–September 2009 to the Department of Clinical Microbiology at Linköping University Hospital were included. The laboratory is the single provider of clinical microbiology diagnostic services in the 430,000-population county of Östergötland, southern Sweden, and is accredited by the Swedish SWEDAC authority and participates in the UK NEQAS quality assessment scheme. There were samples from 92 patients, with dogs implicated in 55, cats in 32, snakes in two and one each for hedgehog, rabbit and rat.

### Reference strains

*Capnocytophaga canimorsus* (CCUG (Culture Collection, University of Göteborg, Sweden) 42590 and CCUG 42737), *Neisseria weaveri* (CCUG 54397), CDC group NO-1 (CCUG 56474), *Neisseria zoodegmatis* (CCUG 56692) and *Sphingomonas ginsenosidimutans* (CCUG 60876) were used as references for growth and 16S rDNA sequencing, and *Streptococcus pneumoniae* (ATCC 49619), *Haemophilus influenzae* (NCTC 8468) and *Neisseria meningitidis* (ATCC 13077) were used for the MIC determinations.

### Bacterial culture

The wound swab samples were stored in a refrigerator at the doctor’s centre and transported under refrigeration to the laboratory and plated on the afternoon of the same day. Samples taken after office hours were stored in a refrigerator and sent the next morning. The transport medium was Copan Transystem® Amies agar gel with charcoal. The samples were spread on culture medium upon arrival to the laboratory, according to standard laboratory procedures using Petri dishes with: GC Agar (Acumedia) supplemented with Soluble Haemoglobin Powder (Oxoid) 10 g/L, in-house IsoVitaleX 10 mL/L and inactivated horse serum 100 mL/L; Columbia Blood Agar Base (Acumedia) supplemented with Tryptophan (Duchefa) 0.1 g/L and citrated horse blood 60 mL/L; and CLED agar (Oxoid Microbiology Products). Anaerobic cultures were done on pre-reduced blood agar as above. The dishes were incubated at 36 °C for 2 days, both aerobically, in a moist 5 % CO_2_ atmosphere, and anaerobically. In addition, all samples were subjected to a protocol designed for the isolation of fastidious GNOP. In this GNOP protocol, samples were spread on a GC agar Petri dish containing Acumedia GC agar (7104A) supplemented with 2 % soluble haemoglobin LP0053 (Oxoid®), IsoVitaleX™ enrichment and 10 % inactivated horse serum, and on an MH-F agar Petri dish containing BBL Müller Hinton II™ agar supplemented with 60 mL defibrinated horse blood and 4 mL NAD solution per litre. The two Petri dishes were incubated in a moist 5 % O_2_, 10 % CO_2_ and 85 % N_2_ atmosphere at 36 °C. The cultures were inspected at days 1, 2, 3 and 5. For samples with no GNOP-suggestive colonies, a final check was made on day 7. In samples with heavy growth, a re-streak was performed onto the same GC agar Petri dish and then supplemented with gentamicin (4 mg/L) and trimethoprim (4 mg/L) in order to enhance the detection of *Capnocytophaga* sp. After Gram staining and testing for oxidase (BBL™ DrySlide™ Oxidase), Gram-negative and oxidase-positive finds were re-streaked for purity. Isolates were kept at −70 °C for up to 1 year until 16S rDNA amplification and sequencing [8], and MIC determination were performed. If found to be appropriate, the DNA results (notably *Eikenella corrodens*) were confirmed by biochemistry.

### 16S rDNA gene sequencing

In order to ensure that multiple 16S rDNA PCR amplifications and subsequent amplicon sequencing assays will represent the same isolate, sufficient bacterial DNA was produced by means of whole genome amplification using multiple displacement amplification. Bacterial colonies were picked with a sterile plastic loop and dispersed, lysed and amplified using an Illustra GenomiPhi V2 DNA Amplification Kit (GE Healthcare Bio-Sciences, Uppsala, Sweden). After completion of the reaction, 80 μL ultra-pure water was added to each tube and 1 μL was used as template in downstream applications. Near full-length 16S rDNA PCR amplification was carried out using an M13 sequence-tagged sense primer, an antisense primer and an Applied Biosystems 2720 Thermal Cycler (Applied Biosystems, Foster City, CA, USA) as previously described [9]. This yields an approximately 1400-bp amplicon, including the 16S rDNA variable V1–V8 regions. DNA sequence analysis of 16S rDNA PCR amplicons was carried out using an M13 uni (−21) sequencing primer by a customer DNA sequencing service (Eurofins MWG Operon GmbH, Ebersberg, Germany). Prior to DNA sequencing, PCR amplicons were purified using an Illustra GFX PCR DNA and Gel Band Purification Kit (GE Healthcare Bio-Sciences, Uppsala, Sweden). Conditions throughout were as described in detail previously [9]. The obtained DNA sequences (600–800 bp) were analysed and compared using SeqMatch at the Ribosomal Database Project (http://rdp.cme.msu.edu/seqmatch/seqmatch_intro.jsp) and the leBIBI database (http://umr5558-sud-str1.univ-lyon1.fr/lebibi/lebibi.cgi). The V1–V4 regions have been shown to be particularly suitable for species-level identification of bacteria [10], and these regions are covered by our sequence analysis. The instructions from the respective databases were followed when deciding the species. In some cases, the species level could not be reached and, instead, the genus level was chosen.

### Antibiotic susceptibility testing and interpretation

MIC values were determined with the Etest® (bioMérieux) for ampicillin, benzylpenicillin, oxacillin, cefotaxime, meropenem, gentamicin, clindamycin, erythromycin, tetracycline, trimethoprim–sulphamethoxazole and ciprofloxacin. Pure antibiotic substances were used, except trimethoprim–sulphmethoxazole, to serve as group representatives. A penicillin V (pcV) 10-μg disc (Oxoid) was used for disc diffusion. For GNOP strains retrieved using the MH-F agar, the MICs were determined on that medium and the MICs of the remaining isolates were determined on the GC agar. The reference strains were tested for antibiotic sensitivity using the GC dish, with no apparent difference in the result from the MH-N medium. β-Lactamase production was tested with Oxoid nitrocefin discs.

## Results

In total, there were 92 samples and, of these, eight showed no bacterial growth with either the routine or the GNOP protocol. In two of these negative samples, the patients had been antibiotic-treated and in two cases, the wounds were almost healed. In the 63 samples with GNOP, there was, in addition to GNOP, the following findings: *S. aureus* in nine, coagulase-negative staphylococci in two, coagulase-negative staphylococci and *Enterobacter* in one, coagulase-negative staphylococci and α-streptococci in one. Among the 21 culture-positive samples with no GNOP retrieved, there were the following isolates: *S. aureus* in nine, coagulase-negative staphylococci in one, *Bacteroides fragilis* in one and, finally, ten samples showed a diverse human skin flora regarded to be of less clinical relevance. No GNOP was found in the wounds caused by the hedgehog or the snakes.

Following phenotypic analysis (colony morphology, oxidase testing and Gram stain), 220 isolates were judged to represent GNOP and selected for 16S rDNA sequencing. There were more GNOP isolates from dog-caused wounds (2.2 per sample) as compared with cats (1.5 per sample) (Table 1). Because of the limited clinical information available, we have used an indirect estimate of infection severity, by implying that a sample from a hospital ward reflects higher severity than the out-patient setting. The difference between dogs and cats was even higher in samples (*n* = 26) originating from hospitalised patients, with 2.7 GNOP for dogs and 1.3 for cats. In contrast, among the out-patient samples (*n* = 35) there were similar rates of GNOP isolation for dogs (1.7) and cats (1.6). *Pasteurella multocida* was the most common species (found in 7 samples) in patients requiring hospitalization, but it was isolated also from 16 out-patients. Notably, some species were found more often in hospitalised as compared with out-patient samples; they include *Frederiksenia canicola* (5 and 0), *N. weaveri* (6 and 5), *Bergeyella zoohelcum* (6 and 3) and *C. canimorsus* (4 and 1).

**Table 1:** Number of GNOP isolates from samples of hospitalised and non-hospitalised patients, serving as indicators of the severity of the infection

Sample data	Animal causing the wound		
	Dog	Cat	Other^a^
Total samples	37	24	2
GNOP isolates	82	36	3
GNOP isolates/sample	2.2	1.5	1.5
Hospital samples	19	7	1
GNOP isolates	51	9	2
GNOP isolates/sample	2.7	1.3	2
Out-patient samples	18	17	1
GNOP isolates	31	27	1
GNOP isolates/sample	1.7	1.6	1

*GNOP* Gram-negative oxidase-positive bacteria

^a^One rabbit hospital sample and one rat out-patient sample

Three bacterial families, Pasteurellaceae, Neisseriaceae and Flavobacteriaceae, clearly dominated, with 101 out of the total of 118 GNOP isolates in dog- and cat-caused wounds (Table 2). As expected, among the Pasteurellaceae, most isolates were *P. multocida*, but 13 other species were also detected, in both the dog and the cat groups. Whereas *P. multocida* were mostly isolated from cats in out-patient settings, most of the other species in this family were isolated from dogs in hospitalised patients. *Neisseria weaveri* represented a majority within the Neisseriaceae family, but 18 isolates of seven other species were also detected. The Flavobacteriaceae family with six species showed the highest fraction of hospitalised patients (74 %), with relatively few isolates of *C. canimorsus* (*n* = 6) and with the less-known pathogenic *B. zoohelcum* as the most frequent finding (9 of 19). From the rat bite wound, *Pasteurella pneumotropica* was isolated and from the rabbit-caused wound, *Actinobacillus capsulatus* and *B. zoohelcum* were isolated.

**Table 2:** Family and species assignment of the isolated GNOP findings from dog- and cat-caused wounds

Family	Species	Dog	Cat	Hospital	Out-patient
Pasteurellaceae	*Actinobacillus* sp.		1		1
	*Aggregatibacter aphrophilus*	1			1
	*Bibersteinia* sp.	1		1	
	*Bibersteinia trehalosi*	1		1	
	*Frederiksenia canicola*	5		5	
	*Haemophilus haemoglobinophilus*	2		2	
	*Haemophilus parainfluenzae*	1			1
	*Haemophilus* sp.	2		1	1
	*Pasteurella canis*	8		2	6
	*Pasteurella dagmatis*	3		1	2
	*Pasteurella mairii*		1		1
	*Pasteurella multocida*	3	20	7	16
	*Pasteurella pneumotropica*		2	1	1
	*Pasteurella stomatis*	2		2	
Pasteurellaceae, total		29	24	23	30
Neisseriaceae	*Eikenella corrodens*	3		1	2
	*Neisseria canis*	4		1	3
	*Neisseria dentiae*	1		1	
	*Neisseria flavescens*	1		1	
	*Neisseria shayeganii*	1	1	1	1
	*Neisseria wadsworthii*		3	1	2
	*Neisseria weaveri*	11		6	5
	*Neisseria zoodegmatis*	3	1	2	2
Neisseriaceae, total		24	5	14	15
Flavobacteriaceae	*Bergeyella zoohelcum*	7	2	6	3
	*Capnocytophaga canimorsus*	4	1	4	1
	*Capnocytophaga cynodegmi*	1		1	
	*Cellulophaga lytica*	1			1
	*Flavobacterium ceti*	2		2	
	*Flavobacterium* sp.	1		1	
Flavobacteriaceae, total		16	3	14	5
Comamonadaceae	*Comamonas* sp.	1			1
	*Xenophilus azovorans*	1		1	
Moraxellaceae	*Enhydrobacter aerosaccus*		1	1	
Xanthomonadaceae	*Luteimonas terricola*	3		2	1
	*Thermomonas haemolytica*	1		1	
No identification		1		1	
Group 2 designated isolates^a^					
Burkholderiaceae	*Ralstonia pickettii*	5	1	2	4
Caulobacteraceae	*Caulobacter vibrioides*	1			1
Oxalobacteriaceae	*Naxibacter haematophilus*		1	1	
Pseudomonadaceae	*Pseudomonas putida*		1		1

The hospital and out-patient data serve as indicators of the severity of the infection

*GNOP* Gram-negative oxidase-positive bacteria

^a^Arbitrarily designed group of species with possibly only little clinical relevance

Although the pathogenicity for many of the isolated species listed in Table 2 are not well known, we made an attempt to define a group unlikely to be of clinical significance, comprising a total of nine isolates (called group 2) and, out of these, *Ralstonia pickettii* was most prevalent, with six isolates. This group 2 also had a different antibiotic profile from the bulk of the observed GNOP in group 1 (Table 3). Most isolates in group 2 were β-lactamase-producers and resistant to commonly recommended penicillins, but displaying MIC values indicating susceptibility to tetracyclines, trimethoprim–sulphamethoxazole and ciprofloxacin (Table 3).

**Table 3:** MIC_90_, MIC range and a proposal for the interpretation of percentage susceptibility for the antibiotics tested based on the European Committee on Antimicrobial Susceptibility Testing (EUCAST) breakpoints of GNOP isolates

Antibiotic	Group 1 GNOP (*n* = 112)				Group 2 GNOP (*n* = 9)	
	MIC_90_ (mg/L)	MIC range (mg/L)	Susceptible breakpoint (mg/L)	Susceptible (%)	MIC_90_ (mg/L)	MIC range (mg/L)
Ampicillin^a^	0.38	0.016–1	≤1	100	>256	4 to >256
Penicillin G^a^	0.38	0.016–6	≤0.5	91	>256	8 to >256
Oxacillin^b^	96	0.023 to >256	None	–	>256	16 to >256
Cefotaxime^a^	0.094	0.016–0.75	≤0.03	80	2	0.5–32
Meropenem^c^	0.064	0.002–0.19	≤0.5	100	32	0.032 to >32
Gentamicin^b^	64	0.094 to >256	≤1	18	>256	0.19 to >256
Clindamycin^b^	32	0.016 to >256	≤0.25	17	>256	6 to >256
Erythromycin^c^	4	0.016–16	≤0.25	12	16	0.38–96
Tetracycline^c^	0.5	0.016–3	≤1	97	1.5	0.125–6
Trimethoprim–sulphamethoxazole^a^	0.5	0.004 to >32	≤0.25	85	>32	0.032 to >32
Ciprofloxacin^a^	0.094	0.002–0.125	≤0.06	87	0.38	0.064 to >32
Antibiotic		Range (mm)	Susceptible breakpoint (mm)	Susceptible (%)		Range (mm)
Penicillin V^d^		6–51	≥25 mm	74		6–9

*MIC*_*90*_ Minimum inhibitory concentration required to inhibit the growth of 90 % of the isolates; *GNOP* Gram-negative oxidase-positive bacteria

^a^Interpretive susceptible criteria, EUCAST breakpoint tables for interpretation of MICs and zone diameters. Version 6.0, 2016. http://www.eucast.org (EUCAST 6.0), *Pasteurella multocida*

^b^Interpretive susceptible criteria, EUCAST 6.0, *Staphylococcus* spp.

^c^Interpretive susceptible criteria, EUCAST 6.0, *Moraxella catarrhalis*

^d^In the past, a disc diffusion zone of ≥25 mm was interpreted as susceptible

In some samples, there were two or more isolates with identical 16S rDNA species assignment; they were regarded to represent a single strain and were scored as one isolate. After this correction for doublets, 121 isolates were judged as true and unique GNOP findings, and subjected to MIC determination. One isolate each of *Pasteurella dagmatis* and *C. canimorsus* did not survive re-cultivation and no MIC results could be obtained.

The ranges of MIC values for all GNOP isolates belonging to species groups 1 and 2 are given in Table 3. Species-related MIC breakpoints are not available for all species belonging to group 1, but a proposal for the interpretation of percentage susceptibility for the antibiotics tested based on the European Committee on Antimicrobial Susceptibility Testing (EUCAST) breakpoints, version 6.0 [11] is given in Table 3. The number of group 2 isolates was too small for the interpretation of the percentage of susceptibility.

All isolates belonging to group 1 were susceptible to ampicillin and meropenem, >90 % of the isolates were susceptible to penicillin G and tetracycline, and ≥80 % to cefotaxime, ciprofloxacin and trimethoprim–sulphamethoxazole. Penicillin V is an oral alternative often used for the treatment of uncomplicated skin infections. Penicillin V was tested with agar disc diffusion but has no established susceptibility breakpoints. In the past, a disc diffusion zone of ≥25 mm was interpreted as susceptible. Using this breakpoint, 74 % of the group 1 isolates were susceptible to penicillin V, while none among the group 2 isolates had a zone >9 mm.

## Discussion

The main finding in this study is that a wide spectrum of bacteria belonging to the oral flora of animals (mostly cat and dogs), dominated by GNOP, plays a more prominent role in animal-caused wound infections than previously recognised [2–4]. GNOP were isolated from 68 % of the samples, while *S. aureus*, β-haemolytic streptococci group G and anaerobes were isolated in 22 %. A number of factors may contribute to the difference between our data and those previously reported. The present study is based on a consecutive, prospective series of all animal-caused wound samples submitted to a laboratory being the sole provider of clinical microbiology services in a defined area of Sweden. Our material, therefore, comprises patients visiting a general practitioner or an emergency department, as well as those in a hospital ward, and the wounds did have signs of not only an injury but also an infection. This forms a contrast to earlier data, which were derived from either case reports or patient series from emergency departments, often representing more severe cases and not necessarily those with an infection [1, 2, 12]. Many infections have been described in other studies as abscesses, which may explain the high fraction of *S. aureus* and anaerobes in those studies. It is customary in Sweden to seek medical advice early in case of an injury or when signs of infection appear. This might be one reason for the low prevalence of abscess-forming wounds. A diagnostic tradition to focus on *Bacteroides* spp. group, and pay less attention to other anaerobes, may have limited our anaerobe score [13, 14].

Another novel aspect of our study is the wide GNOP repertoire covering more than 30 species, many not appearing in previous reports of infections in animal-caused human wounds. However, this should not be surprising, because the current literature, including the American Society for Microbiology (ASM) Clinical Manual of Microbiology and the latest Centers for Disease Control and Prevention (CDC) Morbidity and Mortality Weekly Report (MMWR) [12], refers to results mostly collected more than 25 years ago [3, 4, 7, 15]. Since that time, several new GNOP species have been named [16], probably due to a combination of improved isolation and identification (e.g. 16S rDNA) methodology. One example is the newly characterised *Pasteurella* family member *Frederiksenia canicola* [16, 17]. In fact, many (11 of the total of 37) of the GNOP species found by us are not listed in the database of the phenotypic matrix-assisted laser desorption/ionisation time-of-flight (MALDI-TOF) procedure now widely used in clinical microbiology laboratories for species identification. Our results may lead to the inclusion in the MALDI-TOF database of the GNOP species reported by us. In this way, our 16S rDNA sequencing results may lead to improved clinical diagnostic services. Based on our experience from diagnostic work in Scandinavia, it is our impression that a GNOP other than *P. multocida* isolated earlier was often perceived as a slowly growing coryneform or a *Neisseria* family member, being reported to the clinician as a ‘member of human skin flora unlikely to be of clinical significance.’

The antibiotics studied by us were selected as they are in common use and are representatives of antibiotic groups, and have relevance for developing as well as industrialised countries. Pure antibiotic substances were chosen, except for the combination of trimethoprim–sulphamethoxazole, where the components appear in fixed compositions. Our observation of ampicillin susceptibility among essentially the entire and wide repertoire of GNOP, and notably including all of the *Pasteurella* and *Neisseria* isolates, coupled with a relatively low incidence of potentially ampicillin-resistant bacteria, such as *S. aureus* (20 %; 18 of 92 wounds), can have a major clinical relevance. Although it should be acknowledged that we have limited information on the degree of pathogenicity for many GNOP, it can be noted that high ampicillin resistance among GNOP was restricted to strains considered to be relevant only in an immunodeficiency setting, and which were allocated by us to a limited clinical relevance ‘group 2’ of Tables 2 and 3. This is illustrated by a recent report on opportunistic infections caused by *R. pickettii*, representing six out of the total of our nine isolates in the GNOP group 2 [18]. According to our experience, group 2 isolates appear in patients suffering from more than week-old burn injuries or with an impaired immune system, being in agreement with the literature. This observation of ampicillin susceptibility is important because it supports the empiric use of this drug and its analogues to cover GNOP. As *S. aureus* is also prevalent in animal-caused wound infections, a second antibiotic may be added, or amoxicillin in combination with the penicillinase inhibitor clavulanic acid may be an alternative, as has been recommended in both Europe [15] and the United States [19]. Most isolates were also susceptible to the bacteriostatic tetracycline, whereas in a serious infection, a bactericidal agent should be preferred. A disadvantage with trimethoprim–sulphamethoxazole is that *C. canimorsus*, one of the potentially fatal ‘bite-species,’ is resistant. For the penicillin-allergic patient, ciprofloxacin, cefotaxime and meropenem are bactericidal alternatives. Another important finding in this study is that the common use of oxacillin or clindamycin, in combination with an aminoglycoside in serious infections, cannot be expected to be effective in most animal-caused wounds, as also shown in earlier data [5, 6]. Our findings emphasise the need to cover GNOP, and not only common wound pathogens, when treating an animal-caused wound.
